# Investigation of Depression in Greek Patients with Diabetic Peripheral Neuropathy

**DOI:** 10.5539/gjhs.v5n5p107

**Published:** 2013-06-16

**Authors:** Maria Rekleiti, Pavlos Sarafis, Maria Saridi, Aikaterini Toska, Chrysovaladis Mellos, Kyriakos Souliotis, Maria Tsironi

**Affiliations:** 1 Faculty of Human Movement and Quality of Life Sciences, Department of Nursing, University of Peloponnese, Sparti, Greece; 2 Faculty of Nursing, Technological Educational Institute, Lamia, Greece; 3 General Hospital of Korinthos, Korinthos, Greece; 4 Faculty of Social Sciences, Department of Social and Educational Policy, University of Peloponnese, Korinthos, Greece

**Keywords:** diabetes, diabetic neuropathy, BMI, overweight, obesity, central obesity, depression

## Abstract

**Background:**

Considerable studies directly connect the complications in diabetic patients, and especially peripheral neuropathy, with the emergence of depression. Neuropathetic pain may deteriorate the general health status of the diabetic patient and glycaemic regulation.

**Purpose:**

The purpose of this study was to investigate the appearance and degree of diabetic peripheral neuropathy and its correlation with depression, with other parameters of the disease and also duration.

**Methods:**

57 diabetic patients participated with diagnosed diabetic peripheral neuropathy (male n=27, female n= 30, mean of age 72.7±6.35 years). The first part of Michigan Neuropathy Screening Instrument and the Zung Depression Rating Scale were used as tools for our study. Data was analysed with the SPSS 18.0 statistic program.

**Results:**

57.9% of the patients were overweight, 35.1% were obese and only 7% were within normal weight range. The BMI findings between the two genders indicate that male participants are more often obese than females. Women surpassed men in the category of overweight patients (p<0.05). The score based on MNSI was high and between 3 to 12 (mean average of 8.19±2.60 with 8 as intermediate rate). Almost 60% of patients had severe neuropathy, only 2 were found with mild symptoms and the rest had moderate neuropathtic symptoms, based on the score summary from the questionnaire. Investigating in detail the relation of diabetic neuropathy and depression, it derives that a high degree of diabetic neuropathy is related with high score of depression [*F*(3.160)=9.821, *p*=0.001]. Moderate and severe neuropathy was found with almost the same levels of depression.

**Conclusions:**

The correlation between diabetic neuropathy and depression is confirmed, while a very high depression rate was found in patients with severe neuropathy. The issue needs further study by using common instruments to obtain comparative results from the scientific community.

## 1. Introduction

Diabetic neuropathy is an umbrella term that covers several clinical syndromes, which affect separate territories of the nervous system, solely or combined together, and is the most problematic of all diabetes complications and leads analogically to larger morbidity and mortality (Vinik et al., 2000; Vileikyte et al., 2005). It is estimated to affect 24% of diabetic patients (Schmader, 2000; Herman et al., 2012). This type of neuropathy is the most frequent in developed countries and increases hospital admissions more than all diabetes complications together (Polikandrioti & Kalogianni, 2009; Vileikyte et al., 2009).

Among the complications of diabetes, the epidemiology of diabetic neuropathy is the less studied, because of insufficient patient choices, incomplete usage of valid instruments and absence of common acceptable staging. In the United States, approximately 70% of diabetic patients present mild to severe neuropathy. Among the patients who visit an outpatient diabetes clinic, 25% complain about symptoms, 50% are found with neuropathy based on the Achilles tendon reflex or pallesthesia control (**a feeling of vibration when examined with a** tuning fork), while almost 90% of them have positive results on more specialised tests (Vinik et al., 2000; Kanjii et al., 2010). In Europe, the results show that 35% of diabetic patients have neuropathy based on symptoms and clinical findings (Wetering et al., 2010; Rekleiti et al., 2012). A recent phone study, involving the whole population of France, indicated prevalence of diabetic neuropathy as high as 11% (Wu et al., 2006).

Regarding the epidemiology of different forms, it seems like diabetic polyneuropahty is the most frequent in type 1 diabetes mellitus patients, as almost 50% of the patients are affected (Martin et al., 2010; Louraki et al., 2012). Mononeuropathy of the medium carpal tunnel syndrome is second most often and it is followed by visceral autonomic neuropathy and other nerve damage. In type 2 diabetes, diabetic polyneuropathy is the most frequent, followed by mononeuropathy of the medium nerve, visceral autonomic neuropathy and other diabetic neuropathies (Duby et al., 2004; Martin et al., 2012).

Peripheral symmetric polyneuropathy is the most frequent and widely recognised form of diabetic neuropathy. The onset is usually insidious because of a non-adequate glycaemic control backround and/or a long history of diabetes, but occasionally it can be fast after stress or the start of antidiabetic treatment and, once established, it is generally non-reversible (Wetering et al., 2010). The dysfunction of minor nerve fibers usually occur early and often exists without the presence of objective clinical findings or electrophysiological indications of nerve damage (Ribas et al., 2012). It emerges under the following forms: nerve fiber neuropathy (acute and chronic painful neuropathy), large fibre neuropathy and mixed neuropathy.

Several studies directly connect the development of complications in diabetic patients, particularly neuropathy, with depression (Vileikyte et al., 2009; Jain et al., 2011). It has also been observed an increase in depressive symptoms when diabetes complications increase in intensity and number (DeGroot et al., 2001; Herman et al., 2012). Neuropathy's severe pain can deteriorate the diabetic patients’ health status and their glycaemic control. Depressive symptoms which occur as a result of pain (insomnia, weight loss) affect the patients’ quality of life, since they face difficulties coping with their social role and satisfying their personal needs (Yoshida et al., 2009).

The purpose of this study was to investigate the appearance and degree of diabetic peripheral neuropathy and the correlation with depression, other parameters of the disease and duration.

## 2. Methods

The reference population was patients with diabetic peripheral neuropathy as a diagnosed chronic complication, which were monitored at an outpatient diabetic clinic. 57 diabetic patients were considered with diagnosed diabetic peripheral neuropathy, from which 27 were male and 30 female, with mean of age 72.7±6.35 years.

### 2.1 Instruments

The questionnaire comprised of three sections: the first section included a questionnaire, designed by the authors, regarding demographics and body measurements, which had previously (Rekleiti et al., 2012) been standardized on a sample of diabetic patients (n=164). The second section included the first part of the Michigan Neuropathy Screening Instrument (MNSI), which is the first stage of the Michigan Neuropathy Program (Feldman et al., 1994). It includes a questionnaire regarding the main subjective complaints for peripheral neuropathy, such as pain, tingling, causalgia, paresthesias, numbness, cramps, skin dryness and other - deteriorating or not- factors, the location and duration of symptoms, in relation with walking, resting and sleeping. The severity of neuropathy was estimated by adding the participants’ scores on the relevant questionnaire items. The participants’ scores fell into the following categories, according to the neuropathy ranges employed by Georgopoulou et al. (2002): neuropathy-free (score: 0-2), mild neuropathy (3-4), moderated neuropathy (5-6), severe neuropathy (7 or higher).

The third section included Zung's Depression Rating Scale (ZDRS) which had 20 questions regarding the evaluation of several emotional, psychological and physical symptoms (Zung, 1965; Foundoulakis et al., 2001). The patient clarifies the frequency of a specific symptom, each answer corresponds to a score and in the end the total score allows us to evaluate the symptoms of depression. The severity of depression on Zung's SDS is estimated with the formula


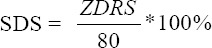


where ZDRS stands for ‘Zung Depression Rating Scale’. The participants’ scores fell into the following categories: normal emotional status (SDS < 55), borderline Depression (55 ≤ SDS < 62), mild depression (62 ≤ SDS < 74), moderate depression (74 ≤ SDS < 86), severe depression (86 ≤ SDS).

### 2.2 Ethics

The approval of scientific councils of the hospitals in which our research took place, and permission from the directors of diabetes clinics was requested. Before the beginning of the interview, the volunteered patients where fully informed. The proper function of the clinics was never disrupted and there was no additional economic burden for the patients or the hospitals. Anonymity of the participants and confidentiality of information were strictly observed.

### 2.3 Statistical Analysis

The data was recorded and analysed by SPSS 18.0 statistical software. Student's *t*-test was used for comparison of means among the two different groups (for example: men vs women, etc.). In order to make assessments among two quantitative variables we used Pearson's correlation coefficient *r*, while the *point biserial* correlation coefficient (*r*pb) was used for dichotomic and quantitative variables.

## 3. Results

**Figure 1:**
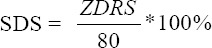
Percentages of BMI according to sex

The sample consisted of 57 diabetic patients with diagnosed diabetic peripheral neuropathy, from which 27 were male and 30 female, with mean of age 72.7±6.35 years. As far as education levels were concerned, 75% of our sample had graduated from elementary school, 12% had graduated from secondary education (high school), and 13% had higher (university level) education. Considering the B.M.I. (Body Mass Index - kg/m^2^) of the participants, 57.6% of the patients were overweight, 35.4% obese and only 7% were within normal weight range. The “underweight” category had no representatives. The BMI findings between the two genders indicate that male participants are more often obese than females. Women surpassed men in the category of overweight patients, as seen in Figure 1 (p< 0.05).

**Figure 2:**
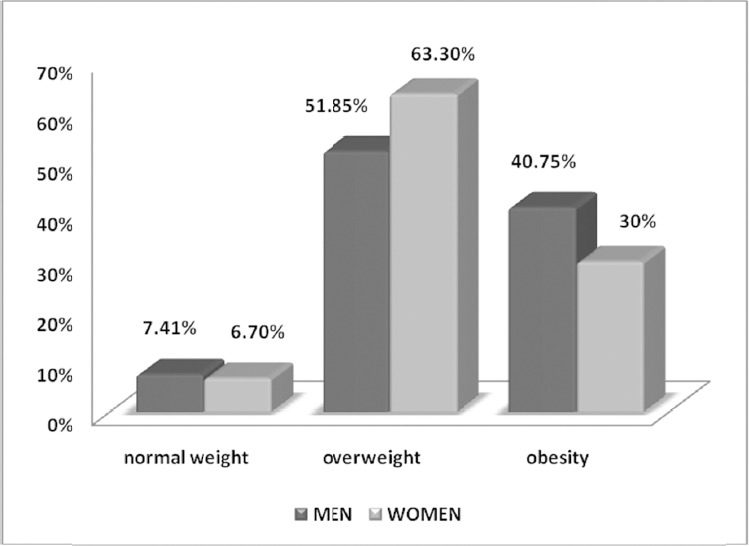
Percentages of central obesity according to sex

The vast majority of men and women had central obesity, based on its definition that for men waist circumference had to be more than 102cm, and for women more than 88cm, as seen in Figure 2.

**Figure 3:**
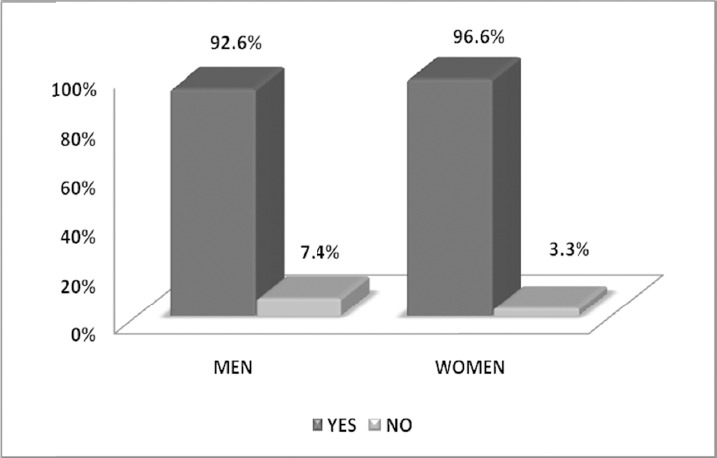
Percentages of diabetic neuropathy

The score based on MNSI was high ranging between 3 to 12 (mean average of 8.19±2.60 with 8 as intermediate rate). 2/3 of the patients had severe neuropathy, only 2 were found with mild symptoms and the rest had moderate neuropathy symptoms, based on the score of the questionnaire, as seen in Figure 3. All patients complained about leg numbness and pain during walking, while none of them had resorted to amputation. Details of the participants’ answers are presented in Table 1.

**Table 1:** Participants’ responses to MNSI questionnaire

Questions	“YES” Answers	

	n	%
Are you legs and/or feet numb?	57	**100%**
Do you ever have any burning pain in your legs and/or feet?	51	89.5%
Are your feet too sensitive to touch?	32	56.1%
Do you get muscle cramps in your legs and/or feet?*	50	87.7%
Do you ever have any prickling feelings in your legs or feet?	55	**96.5%**
Does it hurt when bed covers touch your skin?	29	50.9%
When you get into the tub or shower, are you able to tell the hot water from the cold water?++	34	59.6%
Have you ever had an open sore on your foot?	19	33.3%
Has your doctor ever told you that you have diabetic neuropathy?	45	78.9%
Do you feel weak all over most of the time?*	38	66.7%
Are your symptoms worse at night?	52	**91.2%**
Do your legs hurt when you walk?	57	**100%**
Are you able to sense your feet when you walk?++	45	59.6%
Is the skin on your feet so dry that it cracks open?	24	42.1%
Have you ever had an amputation?	0	**0%**

* Those symptoms are not specific to diabetic neuropathy, but point towards circulatory problems and general weakness respectively. Although they are included in the MNSI tool, they are not taken under consideration at the scoring stage.

++ Inverse-posed questions that at scoring “No” answers are counted as neuropathy symptoms.

Participants were assessed for peripheral neuropathy using the MNSI and for depression levels using the ZDRS. Both instruments yield scores that can be interrelated.

MNSI had a statistically significant correlation [*r*=0.358, *p*=0.001] with depression scores in Zung's questionnaire. Investigating with detail the relation between diabetic neuropathy and depression, it derives that high diabetic neuropathy is related with high incidence of depression [*F*(3.160)= 9.821, *p*=0.001]. Moderate and severe neuropathy was linked to almost the same levels of depression.

The correlation (point biserial correlation) of every MNSI question separated from the depression questionnaire score can be seen in Table 2. None of the individual questions had had an important statistic correlation, although they contributed in a positive manner in the appearance of depression [inability to identify temperature (*r*=0.110, p= 0.4140)]. For leg numbness, pain while walking and amputation it wasn’t possible to calculate the correlation factor, because the first two symptoms were present in all patients and amputation in no one.

**Table 2:** MNSI questions correlated to total scores of the depression questionnaire

Symptom	*Point biserial r*	*P<0.05*
Feeling of weakness*	0.213	0.112
Inability to identify temperature	0.110	0.414
Hypoesthesia of the feet	0.105	0.437
foot ulcer	0.095	0.481
Tingling in the legs/feet	0.090	0.506
Foot cramps*	0.076	0.575
Feet sensitive to touch	0.063	0.643
Dry skin	0.060	0.659
Medical diagnosis	0.048	0.723
Burning sensation in the feet	0.041	0.761
Pain from blanket pressure	0.009	0.948
At night symptoms get worse	0.008	0.954
Leg numbness	---	---
Pain while walking	---	---
Amputation	---	---

* Those symptoms have no relation to diabetic neuropathy, but point towards general weakness and circulatory disorders respectively. They are included in the MNSI, but they are not taken into consideration at the total test scores.

## 4. Discussion

In the present study, the sample consisted of diabetic patients with peripheral neuropathy and their demographics and body measurements were recorded. The patients were visiting outpatient clinics. They were asked to fill two questionnaires about neuropathy and depression symptoms. Most of the participants were by far elderly, overweight or obese with high percentage of central obesity in both genders. The total questionnaire scores showed that the majority suffered from severe neuropathy; as far as depression was concerned, it was shown that the more serious the neuropathy symptoms, the more depression symptoms emerged.

A meta-analysis of 27 studies (1975 – 1999) regarding depression in diabetic patients, revealed a constant significant correlation between depression and the complications of diabetes, diabetic neuropathy being the dominant one (DeGroot et al., 2001). An increase of depression symptoms has been observed simultaneously with an increase of diabetic complications in intensity and number, something that has been established by other studies too (Vileikyte et al., 2005; Rekleiti et al., 2012).

The results of the Gore et al. (2005) study, which examined the severity of pain in diabetic patients with neuropathy in relation to anxiety, depression and sleep, revealed that this specific group of patients had severe sleeping disorders in relation with the general population, increased anxiety and depression symptoms, while severe pain caused problems in movement, work, mood, general activities and enjoying life. As the depression symptoms, the severity of pain and sleeping disorder increased, the patients’ physical, emotional and mental functions decreased.

Another study using a large sample (n=484) tried to correlate neuropathy symptoms (pain, instability, loss of sensitivity) with depression symptoms, and high rates of depression were found -something confirmed by the present study as well (Vileikyte et al., 2005). It was found that the main factors related to the emergence of depression symptoms in diabetic patients with neuropathy, are: perceptions about neuropathy risk, poor or insufficient diabetes treatment, and the patients’ altered social self-consciousness, a finding in accordance with a prior study (Herman et al., 2012).

Several other studies seem to confirm our findings, and show high incidence of serious depression in diabetic patients with neuropathy, as well as stress-related symptoms, sleep problems and work-related changes, resulting in poor health, lower quality of life, poor social life and more visits to doctors. Almost half of the diabetic patients with neuropathy are under anti-depression treatment too. Some studies link all of the above to pain severeness, which has a negative impact on the patient's daily life (Tolle et al., 2006; Baron et al., 2009; Wetering et al., 2010).

Ribas et al. (2012) suggest that neuropathy pain results in loss of functionality and lower mobility. When those symptoms emerge, the patients limit their daily mobility and their depression symptoms increase. According to several studies, proper care aims to relieve pain and restore mobility by using several methods, such as laser treatments, to re-establish good glycaemic regulation, and to help patients comply to their treatments, which was also confirmed by our study (Kyriazis et al., 2013).

Finally, according to Black et al. (2003), the bigger the duration of the pain, the more serious the depression symptoms get; as a result, comorbidity is a prognostic marker of further diabetic complications and bad progress that may influence the patient's quality of life and increase mortality rates -a finding present in our study as well.

It is noteworthy that according to Gendelman et al. (2009), a diagnosis of major depressive disorder in diabetic patients with neuropathy is a weaker prognostic marker of mortality, in comparison to a diagnosis of chronic dysthymia - a finding that was not examined in the present study.

A strong point of this study is that this was a prospective study, conducted using an a priori research protocol. A limitation for the study was the fact that patients did not have easy access to the designated Diabetes Clinics, consequently our sample was smaller than the actual number of diabetic patients with neuropathy that were visiting those Clinics. Moreover, a number of patients had to be excluded since we selected our sample using random sampling rules. Finally, the study had to take place in a limited time frame and on a tight budget, since this study was originally part of a thesis submitted by the author.

In conclusion, many studies in patients with diabetic neuropathy are retrospective, in other words they assess data from hospital patient records. Our conclusions might be valid for all out-patient diabetes clinics throughout the country, yet it is not certain whether they describe precisely the general diabetic population with peripheral neuropathy. A genuine epidemiological research should include and investigate a representative sample from a much wider geographical area, in order to come up with more general and valid conclusions. Moreover, using the same tools for assessing neuropathy symptoms and depression could yield powerful results.

## References

[ref1] Baron R, Tölle T. R, Gockel U, Brosz M, Freynhagen R. A (2009). Cross-sectional cohort survey in 2100 patients with painful diabetic neuropathy and postherpetic neuralgia: Differences in demographic data and sensory symptoms. J Pain.

[ref2] Black S. A, Markides K. S, Ray L. A (2003). Depression predicts increased incidence of adverse health outcomes in older mexican americans with type 2 diabetes. Diabetes Care.

[ref3] DeGroot M, Anderson R, Freedland K. E, Clouse R. E, Lustman P. J (2001). Association of depression and diabetes complications: a meta-analysis. Psychosom Med.

[ref4] Duby J. J, Campbell R. K, Setter S. M, White J. R, Rasmussen K. A (2004). Diabetic neuropathy: an intensive review. Am J Health-Syst Pharm.

[ref5] Feldman E. L, Stevens M. J, Thomas P. K, Brown M. B, Canal N, Greene D. A (1994). A practical two-step quantitative clinical and electrophysiological assessment for the diagnosis and staging of diabetic neuropathy. Diabetes Care.

[ref6] Fountoulakis K. N, lacovides A, Samolis S, Kleanthous S, Kaprinis S. G, Kaprinis G. S, Bech P (2001). Reliability, validity and psychometric properties of the Greek translation of the Zung depression rating scale. BMC Psychiatry.

[ref7] Gendelman N, Snell-Bergeon J. K, McFann K, Kinney G, Wadwa R. P, Bishop F, Maahs D. M (2009). Prevalence and correlates of depression in individuals with and without type 1 Diabetes. Diabetes Care.

[ref8] Georgopoulou F, Thireos E, Kalaitzaki K, Houdeloudi E, Aroni A, Vlachogiannis N (2002). The assessment and staging of possible peripheral neuropathy in diabetic patients: the usage/use of an instruments. Primary Health Care.

[ref9] Herman W. H, Pop-Busui R, Braffett B. H, Martin C. L, Cleary P. A, Albers J. W, Feldman E. L, The DCCT/EDIC Research Group (2012). Use of the Michigan Neuropathy Screening Instrument as a measure of distal symmetrical peripheral neuropathy in Type 1 diabetes: results from the Diabetes Control and Complications Trial/Epidemiology of Diabetes Interventions and Complications. Diabet Med.

[ref10] Jain R, Jain S, Raison C. L, Maletic V (2011). Painful diabetic neuropathy is more than pain alone: examining the role of anxiety and depression as mediators and complicators. Curr Diab Rep.

[ref11] Kanji J. N, Anglin R. E, Hunt D. L, Panju A (2010). Does this patient with diabetes have large-fiber peripheral neuropathy?. JAMA.

[ref12] Kyriazis I, Mendrinos D, Rekleiti M, Toska A, Souliotis K, Saridi M (2013). Diabetic patients are often sub-optimally aware about their disease and its treatment. Int J Caring Sci.

[ref13] Louraki M, Karayianni C, Kanaka-Gantenbein C, Katsalouli M, Karavanaki K (2012). Peripheral neuropathy in children with type 1 diabetes. Diabetes Metab.

[ref14] Martin C. L, Waberski B. H, Pop-Busui R, Cleary P. A, Catton S, Albers J. W, Herman W. H, DCCT/EDIC Research Group (2010). Vibration perception threshold as a measure of distal symmetrical peripheral neuropathy in type 1 diabetes: results from the DCCT/EDIC study. Diabetes Care.

[ref15] Polikandrioti M, Kalogianni A (2009). Education on foot care in people with diabetes mellitus, type II. Vima Asklipiou.

[ref16] Rekleiti M, Roupa Z, Kyriazis I, Wozniak G, Saridi M, Kyloudis P (2012). Self-assessment of depression in patients with diabetes mellitus and its correlation with complications. Arch Hell Med.

[ref17] Ribas E. S, Paiva W. S, Pinto N. C, Yeng L. T, Okada M, Fonoff E. T, Teixeira M. J (2012). Use of low intensity laser treatment in neuropathic pain refractory to clinical treatment in amputation stumps. Int J Gen Med.

[ref18] Schmader K. E (2000). Epidemiology and impact on quality of life of postherpetic neuralgia and painful diabetic neuropathy. Clin J Pain.

[ref19] Tölle T, Xu X, Sadosky A. B (2006). Painful diabetic neuropathy: a cross-sectional survey of health state impairment and treatment patterns. J Diab Complic.

[ref20] Vileikyte L, Peyrot M, Gonzalez J. S, Rubin R. R, Garrow A. P, Stickings D, Boulton A. J (2009). Predictors of depressive symptoms in persons with diabetic peripheral neuropathy: a longitudinal study. Diabetologia.

[ref21] Vileikyte L, Leventhal H, Gonzalez J. S, Peyrot M, Rubin R. R, Ulbrecht J. S, Boulton A. J. M (2005). Diabetic Peripheral Neuropathy and Depressive Symptoms: the association revisited. Diabetes Care.

[ref22] Vinik A. I, Park T. S, Stansberry K. B, Pittenger G. R (2000). Diabetic Neuropathies. Diabetologia.

[ref23] Wetering E. J, Lemmens K. M, Nieboer A. P, Huijsman R (2010). Cognitive and behavioral interventions for the management of chronic neuropathic pain in adults - a systematic review. Eur J Pain.

[ref24] Wu E. Q, Borton J, Said G, Le T. K, Kahn E, Garcia-Cerbrian A, Ingelheim D (2006). Prevalence of diabetic peripheral neuropathy and associated pain in french adults. 16th Meeting of the European Neurological Society.

[ref25] Yoshida S, Hirai M, Suzuki S, Awata S, Oka Y (2009). Neuropathy is associated with depression independently of health-related quality of life in Japanese patients with diabetes. Psychiatry Clin Neurosci.

[ref26] Zung W. W. K (1965). A self-rating depression scale. Arch Gen Psychiatry.

